# Editorial: Multi-omics approaches in the study of human disease mechanisms

**DOI:** 10.3389/fbinf.2024.1546680

**Published:** 2025-01-07

**Authors:** Dapeng Wang, Giuseppe Agapito

**Affiliations:** ^1^ Shandong Key Laboratory of Intelligent Oil and Gas Industrial Software, Qingdao Institute of Software, College of Computer Science and Technology, China University of Petroleum (East China), Qingdao, China; ^2^ National Heart and Lung Institute, Imperial College London, London, United Kingdom; ^3^ Department of Law, Economics and Social Sciences, University Magna Græcia, Catanzaro, Italy; ^4^ Data Analytics Research Center, University Magna Græcia, Catanzaro, Italy

**Keywords:** multi-omics, human disease, data integration, bioinformatics, data analysis

With the development and popularity of high-throughput next-generation sequencing technologies, omics approaches gradually become the essential tool of modern biological and medical research, such as genomics, transcriptomics, proteomics and radiomics. In the early years, most studies used single omics to profile the specific type of biological molecules, which can generate inconsistent biomarkers with different rankings across omics types. With the advancement and cost-effectiveness of the omics, high-quality key biomarkers as well as molecular pathways and regulatory networks causatively associated with diseases can be identified through the co-called multi-omics with more than one type of omics (Hasin et al., 2017). In a typical multi-omics study, one would compare disease samples with controls and compare samples with different severities or different progressive stages to explore the disease-specific or stage-specific molecular features pending further experimental verification. The combination of demographic and clinical data with multi-omics data from patients with a specific disease offers a unique opportunity to make full use of cutting-edge artificial intelligence methods including machine learning and deep learning to accumulate knowledge and experience in interdisciplinary research fields (Reel et al., 2021; Ballard et al., 2024). The most informative analysis is through the multi-omics data from the same set of samples with longitudinal information in order to illuminate the time-dependent dynamic disease progression characteristics. For the multifaceted and complex diseases, multi-omics could define groups of patients with distinct endotypes exhibiting heterogeneous treatment responses due to their particular underlying molecular mechanism connecting genotype with phenotype (Tyler and Bunyavanich, 2019). The findings from these studies could inform the early diagnosis, prediction of prognosis and implementation of most appropriate and effective treatment strategies for the disease, leading to the improvement of the quality of life for patients and realisation of personalised medicine. Recently, multi-omics has been extensively employed in the studies of human diseases, including rare diseases, cancers, and other common diseases. For example, it has proven to be instrumental in the prediction of response to treatment in breast cancers (Sammut et al., 2022), the identification of epigenetic changes in human brains with Alzheimer’s disease (AD) (Nativio et al., 2020), and the improvement of diagnostic yield and clinical management of patients with rare diseases (Lunke et al., 2023). The advent of single-cell omics and spatial omics revolutionised our ways of discovering new cell types at enhanced resolution, elucidating the cellular heterogeneity and cell-cell interactions and measuring three-dimensional architecture and organisation of molecular profiles in a whole tissue (Bressan et al., 2023). Even though the integration of multi-omics has shown the powerful performance in the molecular characterisation of human disease aetiology, the data collection, analysis and harmonisation have presented enormous challenges due to the varied development stages of different omics techniques. In particular, sequencing-based transcriptomics has more established and standardised pipelines for both experimental and bioinformatic processes, as compared to other mass spectroscopy-based omics types, such as proteomics and metabolomics. Besides, transcriptomics can cover all human protein-coding genes whereas proteomics can only screen a selection of human proteins, which makes the integration of transcriptomics and proteomics less comprehensive. Furthermore, the complexity and high-dimensional structure of high-volume multi-omics data offers new avenues for the development of mathematical, statistical, computer science and data science approaches, including data housing, management strategies and data visualisation.

The Research Topic on “*Multi-omics approaches in the study of human disease mechanisms*” is comprised of three original research articles and one brief research report. The contributions to this Research Topic explore how the combined use of multiple techniques can help researchers to gain new insights into disease pathogenesis and drug discovery and development. In particular, they present the development of innovative omics data analysis pipelines and methods that improve the ability to interpret complex data sets.

Gene set enrichment analysis is part of the routine analysis of omics studies and efforts have been constantly made to extract the biological insights following the differential expression or abundance analysis between disease group and control group because of the limitations of the existing methods. To account for the uncertainties in the gene set generated by the differential expression analysis of omics studies, Hemandhar Kumar et al. developed bootGSEA, which used bootstrap approach to analyse randomly selected subsets of data and calculated the integrated score based on rank aggregation of bootstrap replicates and multiple datasets. They also devised an evaluation framework to assess the robustness of the analysis by comparing the results with and without a bootstrap step. The application of the method in the transcriptomics data from renal cell carcinoma and transcriptomics and proteomics data from a spinal muscular atrophy (SMA) mouse model demonstrated an increase in the robustness of the analysis with improved biological interpretation and the effectiveness of the new method in the analysis of different datasets from single-omics or multi-omics studies.

Complex diseases usually involve multiple factors from multiple molecular dimensions, and multi-omics analysis could facilitate the systematic investigation of pathogenesis of the diseases. Vacher et al. used machine learning algorithms to establish a classification model of patients with Alzheimer’s disease based on four individual omics domain including SNP, methylation, RNA and proteomics and their combination. The evaluation results suggested that the integration of the four omics datasets provided the best prediction performance than any of the individual datasets and demonstrated the feasibility of using machine learning approaches in the multi-omics datasets. The group of optimal features identified through the multi-omics analysis spanned across the four different omics categories, including those involved in neurodevelopmental pathways and other uncharacterised features with unknown functions.

Association rule mining is one of the powerful tools for elucidating the directional relationship among various genes and discovering the most relevant rules to the disease status. Mallik et al. reported a *MOOVARM* (multi-objective optimized variable cutoff-based association rule mining) framework for identifying the top rules from multi-omics datasets according to multiple and dynamic support thresholds, confidence thresholds and lift thresholds estimated from the integrative analysis of the data. Furthermore, they tested their new method in three different types of omics datasets including gene expression and DNA methylation in high-grade soft tissue sarcomas as well as protein-protein interaction data. The top ranked optimised rule created a signature of three genes (*STAT3*, *TP53*, *MAPK3*), suggesting the potential directional regulatory role of them in the pathogenesis of the disease. Top ten rules identified through *MOOVARM* produced the best overall classification accuracy, as compared to those identified from other two methods, such as *Apriori* and *Eclat*.

To provide a guidance for choosing the appropriate deconvolution methods, Slabowska et al. evaluated the performance of three major methods, namely Cell2location, RCTD, and spatialDWLS, for spatial transcriptomic data from patients with cardiovascular disease and chronic kidney disease, based on the comparison with annotations provided by histologists. All three methods achieved similar accuracies, and they had poor performance on certain cell types, such as endothelial cells. The running time of Cell2location for deconvolution was much longer than that of the other two methods, and Cell2location was able to generate consistent deconvolution results at a smaller reference size. C2L vs. RCTD showed greater similarity for a number of cell types in all three pairwise comparisons.

The research papers in the Research Topic highlight the remarkable achievements in the field and the need for further and continuous improvement in technologies and methodologies for investigating multi-omics data. Integrating different types of complex omics data can revolutionise the current approach to healthcare, enabling more precise and effective interventions for many diseases. In conclusion, this Research Topic showcases the recent developments and promising implementations of bioinformatic methods in multi-omics studies on various human disease types.
